# Skin Diseases and Syphilis

**Published:** 1896-11

**Authors:** 


### SELECTIONS AND ABSTRACTS.

### SKIN DISEASES AND SYPHILIS.

Edited by Dr. M. B. Hutchins.

### The Treatment of Skin Disfigurements by Electrolysis.*

The subject which I have selected is by no means a new one, but it is one about which not much is generally known. The destruction of various abnormal growths by "electricity" has suffered some degree of bad favor through the performances of our enemies "the quacks." That electrolysis has a wide application and a legitimate use I am convinced by my own experience during the past five or six years.

A very good definition of electrolysis is given by "The National Medical Dictionary" as, "The electro-chemical decomposition of a substance."

To do the work outlined in this paper a few galvanic cells, a sponge electrode, a needle-holder, and, for accurate work, a milliamperemeter are necessary; and, to do it properly, some experience.

In most cases the needle should be attached to the negative pole. The current used is weak, usually advised as that from three to seven or eight cells; with a milliamperemeter we find that we get from one-half to five milliamperes, from these, according to the relative position of the electrodes, a greater amount of intervening tissue reducing the current by resistance, a lesser interval increasing it. Again the number of needles used, the manner of touching or grasping the sponge electrode, the wetness of the sponge, all influence the strength of the current.

The strength of current used in the majority of these operations is harmless to the patient, in fact it would not be felt upon an unbroken epidermis. It is intended to get a decomposing, solvent action in most cases, not an action so intense as to have no ad-

*Read before the State Medical Association of Georgia, at Augusta, April, 1896, by M. B. Hutchins, M.D., Clinical Lecturer on Skin Diseases and Syphilis, Atlanta Medical College, Atlanta, Ga.

vantage over caustics and cauteries. There is not even physical warmth in the needle as used.

While many favor a needle-holder without the spring for opening and closing the circuit, I prefer and have long used the one with this attachment, allowing the patient to keep the wet sponge electrode in the hand, or an assistant to hold it in contact with the patient in operations under anæsthesia. An ordinary steel needle, varying in size to meet the requirements, usually suffices, and it is not necessary to use expensive "special" needles. Proper insulation may be used if needed.

When the circuit is first closed there is a feeling of burning and stinging about the needle, but sensation is benumbed in a few seconds. Charlatans claim to use electrolysis painlessly. They can only do so with the aid of anæsthesia, or with an insufficient current.

Local anæsthetics can only be used on a limited area, and their effect is of short duration. Driving cocaine solution into the follicles by "cataphoresis" might be of use. Chloride of ethyl, for freezing and benumbing the part, is useful. Cocaine solution hypodermically does well for small growths.

General anæsthesia cannot be employed in the daily removal of hairs.

In cavernous nævi, "port-wine marks," and other cases which can be finished in an hour or two, or which can be treated at intervals, it is available.

Sometimes, especially where the punctures are very close together, we may get considerable superficial inflammation. A soothing lotion reduces this, however, in a few hours. Scarring cannot occur unless much of the papillary layer, or deeper, is destroyed. I have never produced it where it was undesirable.

A good knowledge of the minute anatomy of the skin and of the disfigurements to be treated is essential to successful work.

Some of the conditions amenable to this treatment are mentioned below.

HYPERTRICHOSIS—For the removal of superfluous hairs, on the face of ladies especially, electrolysis is the only method. Many of the patent hair-removers are excellent stimulants to its growth.

So are pulling out, cutting, and shaving. A lounge, good eyes, a good light, a steady hand, and perseverance are necessary to do the work properly. The patient holds the sponge electrode, positive, the needle is attached to the negative pole. If a steel needle is used attached to the positive pole a black point results, from the deposit of the "black oxide of iron." The needle may be very small for fine hairs, larger for coarse ones.

A current of from one-half to three milliamperes will be sufficient. Remembering resistance, this can easily be managed.

The needle is passed along the side of the hair to the bottom of the follicle, which can be felt, and the circuit closed. Frothing will occur in most follicles, and the circuit can be opened in from five to thirty seconds. The hair then usually comes out with no resistance to the forceps. If it "pulls" reapply the current through the needle in the follicle. Counting the hairs removed is advisable, in view of a later visit from the patient. Those, the papilla of which has not been destroyed will return, and fine hairs, too small for removal, may be stimulated to active growth by the irritation in their vicinity. Other fine hairs grow large in the course of time even, I think, until a woman has no more to develop, regardless of age. An hour is usually long enough to work but I have had to make each operation very much longer in several cases.

It is proper, as Dr. C. W. Allen of New York says, to have a thorough understanding with your patient before you begin, both as to returns and new growth. It is also as well to be frank about the pain; better to have them find it less, rather than more than they anticipated. I have had one case from out of town where it was necessary to remove nearly two thousand five hundred hairs from the chin and mylo-hyoid region in ten days. The patient bore the pain for the result, and there was no scarring. The amount of work still necessary I am not able to report.

MOLES—I have never seen a mole which I thought becoming, and I have never seen an injury result from their treatment. I have removed one by excision, which had become epitheliomatous through a razor-cut; there was no recurrence. Moles with the hairs in them will frequently disappear from the simple effect of the current used in destroying the hairs, but destroying the mole does not always destroy the hairs—they are deeper.

The advantages of electrolysis for the destruction of moles and other small disfigurements are, the absence of bleeding, no superstition about "the knife," no wound to dress, and finally, where the current is not too strong, no scarring. The needle is pushed through the base of the mole, if it is a large raised one, to the skin opposite. The current produces a tallowy blanching of the mole with some frothing around the needle and sometimes through a hair follicle in line. A transfixion at right angles to the first is then made, and as many others, radially, as required by the size of the growth.

Flat moles are best treated by perpendicular punctures to their depth. A sheaf of many needles will save time, but the extra resistance demands more current. A form of atrophy goes on for some weeks after treatment, more marked at and along the needle punctures. Any elevated, remaining, or level pigmentary points can be removed at later sittings, by the perpendicular application of the needle. In most cases it will require a close search, after the end of the treatment, to discover the site of the mole.

WARTS of various kinds so tedious of treatment with the old method, save that of excision with or without cauterization, usually dissolve into froth under the action of electrolysis, being more easily destroyed than the average mole.

EPITHELIOMA.—Remembering the histology of epithelioma—one just beginning, or a single recurrent point should yield to this treatment. A case of mine, of the nose, very rebellious, was finally cured by this means.

Small FIBROMATA, *milia, sebaceous cysts,* in fact any small growth, can be destroyed by electrolysis.

KELOIDS and *hypertrophic scars* are benefited by this measure. Keloid is the chief of recurrers, and excision is usually nullified by re-growth from the cicatrix.

COMEDONES which are persistent may be gotten rid of by the electrolytic destruction of the gland whose orifice it plugs, the current, besides its special action, acting like an irritant injection. The same action takes place in retention cysts. Brocq, of Paris, has followed the same procedure in oily, rosaceous conditions of the nose. ACNE LESIONS, especially the indurate, afford indications for electrolysis.

ANGIOKERATOMA, a warty condition, with dilated vessels, is most successfully treated by this method.

DILATED CUTANEOUS VESSELS, whether they occur in *rosacea, acne rosacea,* about and in scars or as a "*birth-mark,*" yield to this treatment without noticeable scarring, if any.

The so-called "PORT WINE MARKS" require much time for their removal, but perseverance will accomplish it.

The old "NÆVUS ARANEUS" is easily destroyed. In these dilatations of the vessels the needle to the negative pole may be inserted either at right angles or longitudinally to the vessels, or if the dilatation is rounded, larger, directly in the center.

ANGIOMA CAVERNOSUM of considerable size requires a more powerful current, the needle to the positive pole for better coagulation, the current being changed to negative at last to free the needle. Their treatment is very tedious.

The method has been used to remove FRECKLES and other pigmentations.

I have mentioned many conditions which can best be treated by electrolysis, and have only to say—in concluding— that the paper read is based almost entirely upon my own practical experience, not upon pure theory, and that my faith in the method is due both to its results in practice and upon myself.

